# Both diet and *Helicobacter pylori* infection contribute to atherosclerosis in pre- and postmenopausal cynomolgus monkeys

**DOI:** 10.1371/journal.pone.0222001

**Published:** 2019-09-06

**Authors:** Traci L. Testerman, Cristina Semino-Mora, Jennifer A. Cann, Beidi Qiang, Edsel A. Peña, Hui Liu, Cara H. Olsen, Haiying Chen, Susan E. Appt, Jay R. Kaplan, Thomas C. Register, D. Scott Merrell, Andre Dubois

**Affiliations:** 1 Department of Pathology, Microbiology and Immunology, University of South Carolina School of Medicine, Columbia, SC, United States of America; 2 Department of Medicine, Uniformed Services University of the Health Sciences, Bethesda, MD, United States of America; 3 Medimmune, Rockville, MD, United States of America; 4 Department of Mathematics and Statistics, Southern Illinois University at Edwardsville, Edwardsville, IL, United States of America; 5 Department of Statistics, University of South Carolina, Columbia, SC, United States of America; 6 Department of Preventive Medicine and Biostatistics, Uniformed Services University of the Health Sciences, Bethesda, MD, United States of America; 7 Wake Forest University Primate Center, Wake Forest University School of Medicine, Winston-Salem, NC, United States of America; 8 Department of Microbiology and Immunology, Uniformed Services University of the Health Sciences, Bethesda, MD, United States of America; Nagoya University, JAPAN

## Abstract

A number of viruses and bacterial species have been implicated as contributors to atherosclerosis, potentially providing novel pathways for prevention. Epidemiological studies examining the association between *Helicobacter pylori* and cardiovascular disease have yielded variable results and no studies have been conducted in nonhuman primates. In this investigation, we examined the relationship between *H*. *pylori* infection and atherosclerosis development in socially housed, pre- and postmenopausal cynomolgus macaques consuming human-like diets. Ninety-four premenopausal cynomolgus monkeys (*Macaca fascicularis)* were fed for 36 months an atherogenic diet deriving its protein from either casein lactalbumin(CL) or high isoflavone soy (SOY). Animals were then ovariectomized and fed either the same or the alternate diet for an additional 36 months. Iliac artery biopsies were obtained at the time of ovariectomy and iliac and coronary artery sections were examined at the end of the study. Evidence of *H*. *pylori* infection was found in 64% of the monkeys and 46% of animals had live *H*. *pylori* within coronary atheromas as determined by mRNA-specific *in situ* hybridization. There was a significant linear relationship between the densities of gastric and atheroma organisms. *Helicobactor pylori* infection correlated with increased intimal plaque area and thickness at both the premenopausal and postmenopausal time points and regardless of diet (p< 0.01), although animals consuming the SOY diet throughout had the least amount of atherosclerosis. Additionally, plasma lipid profiles, intimal collagen accumulation, ICAM-1, and plaque macrophage densities were adversely affected by *H*. *pylori* infection among animals consuming the CL diet, while the SOY diet had the opposite effect. Plaque measurements were more highly associated with the densities of *cagA-*positive *H*. *pylori* within coronary atheromas than with the densities of gastric organisms, whereas plasma lipid changes were associated with *H*. *pylori* infection, but not *cagA* status. This study provides strong evidence that live *H*. *pylori* infects atheromas, exacerbates atherosclerotic plaque development, and alters plasma lipid profiles independently of diet or hormonal status. Finally, socially subordinate animals relative to their dominant counterparts had a greater prevalence of H. pylori, suggesting a stress effect. The results indicate that early *H*. *pylori* eradication could prevent or delay development of cardiovascular disease.

## Introduction

Prevention of atherosclerosis–the pathobiological process that underlies cardiovascular disease (CVD)–has focused primarily on modifiable risk factors such as smoking, obesity, hypertension, and diet [1]. The hypothesis that chronic infections promote atherosclerosis is more controversial, even though acute infection has been recognized previously as a major contributor to atherosclerosis. For example, in the early 1900s, physicians noted that even young people who died from typhoid fever or other infections frequently had atherosclerotic plaques, whereas those dying from other causes did not [2]. More recently, correlative evidence between atherosclerosis and chronic bacterial and viral infections has been uncovered, or perhaps rediscovered. Potential contributors include periodontal disease, Herpes Simplex virus, Epstein-Barr virus, cytomegalovirus, *Chlamydia pneumoniae*, and *H*. *pylori* [3, 4].

*H*. *pylori* is a Gram negative, spiral or curved bacterium that colonizes the gastric mucosa of humans and other primates. Importantly, *H*. *pylori* is not an acidophile; it can only survive acidic conditions temporarily and must exit the acidic gastric lumen and enter the mucous layer, which has a neutral pH, in order to survive and grow [5–7]. Although *H*. *pylori* is best known for causing peptic ulcers and gastric cancer, it also has been associated with a number of extragastric disease processes, including atherosclerosis (reviewed in [8, 9]). This is not surprising in view of evidence that *H*. *pylor*i colonizes extragastric tissues, as the bacteria have been cultured from the middle ear and liver and detected by immunohistochemistry in skin as well as nasal polyps [10–13]. However, elucidation of a role for *H*. *pylori* in extragastric diseases is complicated by the fact that *H*. *pylori* is genetically heterogeneous and some virulence factors are not universally present. Thus, only certain *H*. *pylori* strains may contribute to specific types of disease pathology. The major virulence factor of *H*. *pylori* is the CagA (Cytotoxin Associated Gene A) cytotoxin that is injected into host cells and alters host cell signaling pathways [14]. *H*. *pylori* strains that possess the *cagA* gene are associated with a greater risk of ulcers and gastric cancer relative to strains that do not [15, 16]; here we assess the degree to which CagA may also contribute to atherosclerosis and, by extension, CVD.

Early epidemiological studies that examined the potential for *H*. *pylori* to contribute to CVD yielded conflicting results [17–20]. However, recent studies increasingly suggest that *H*. *pylori*, and particularly *cagA-*positive *H*. *pylori* strains, may accelerate the development of atherosclerosis and the resulting CVD [21–25]. Finally, although *H*. *pylori* DNA has been detected in atherosclerotic plaques by numerous investigators using PCR[26–30], it remains uncertain whether the bacteria in these plaques are intact or viable.

CVD is the leading cause of death in women over 50 [31]. Improved prevention and treatment strategies have reduced CVD incidence in older individuals, but not among women under age 55 [32]. Premenopausal women have lower rates of CVD than similarly aged men do, yet the role of estrogen in cardioprotection remains unclear [31]. For these reasons, studies in women or suitable female animals are essential for clarifying CVD risk factors in young and older women.

Cynomolgus monkeys (*Macaca fasciularis*) have been used extensively to model the development of atherosclerosis in relation to diet, hormonal conditions, psychosocial factors, and pharmaceutical manipulations [33]. Atherosclerosis in this species closely mimics the pattern observed in human beings, including the relative inhibition of disease progression in reproductively mature females compared to males, the extensive involvement of the coronary and carotid arteries, and the exacerbating effect of traditional risk factors (e.g., hypertension and a diet high in cholesterol and saturated fat) as well as psychosocial stress [34–36]. Finally, responses to a wide variety of pharmaceutical interventions also resemble those observed in people [37].

The purpose of the present study was to determine in this well-established monkey model whether infectious agents–including *H*. *pylori* and a number of viruses–influence atherosclerosis and whether other risk factors, such as diet, reproductive hormones, and psychosocial stress modify any infection-associated effects. Hence, we measured atherosclerosis and plasma lipid composition in pre- and postmenopausal cynomologus macaques following consumption of diets that derived their protein from either animal (casein lactalbumin, CL) or high isoflavone soy isolate (SOY). Most notably we found that *H*. *pylori*, particularly *cagA-*positive *H*. *pylori*, positively correlates with atherosclerosis lesion size and dyslipidemia irrespective of diet treatment, whereas viral infections did not correlate with indices of atherosclerosis extent or severity. Psychosocial stress, as indicated by subordinate social status, was associated with an elevated prevalence of *H*. *pylori*.

## Materials and methods

### Animals and study design

The research described in this manuscript (including the acquisition of monkeys and all protocols for their use) was approved by the Wake Forest School of Medicine Institutional Animal Care and Use Committee (Protocols A97-157, A01-195, and A04-116, encompassing approximately eight years of *in vivo* investigation preceded by a one year run in and a one year close down). Wake Forest is an AALAC accredited institution and all animal care procedures followed the NIH guide and the Principles for the Ethical Treatment of Non-human Primates of the American Society of Primatologists. In addition to the foregoing, the research described here was conducted in accordance with the standard operating procedures of the Wake Forest Environmental Enrichment Program, which involves the provision of cage devices and structures that include at a minimum hanging devices, mirrors, and perches in each pen, and regular surveillance under a behavioral management program designed to identify and mitigate atypical behavior. Finally, all animals were housed continuously in stable social groups–a situation designed to maximize the opportunity to engage in normal, species typical behavior. Macaques were fed twice per day with sufficient food that some remained uneaten.

Ninety-four (94) monkeys were randomized into the treatment conditions for this study. Eighteen (18) died over the following seven years of in-vivo investigation and an additional 2 monkeys became diabetic and had to be removed from the study. This meant that seventy-four monkeys survived until experimental necropsy, which for all cases involved use of sodium pentobarbital to induce euthanasia.

Of the 18 in-study deaths, 2 occurred in year two, 5 in year three, 2 in year four, 6 in year five, 1 in year six, and 2 in year seven. A complete anatomic and histopathologic necropsy was performed on each dead animal and a specific cause of death could be determined in 15 of the 18 cases. Causes of death were varied and included gastric dilation (2 cases), bladder carcinoma, acute cardiac arrest, congestive heart failure, oligodendroglioma, hypoglycemia, hyperglycemia, arterial disease, right atrial thrombosis, and intraoperative hypoxia. There were no deaths due to trauma among the animals in this study. This number of deaths and the development of diabetes is not unusual in a study that persists for almost eight years in-vivo, involves consumption of a human-like diet, and includes a major surgery (oophorectomy) at the midpoint (years four and five).

All surgery and euthanasia was performed under aseptic conditions using sodium pentobarbital anesthesia to minimize suffering. Specifically, this investigation was categorized as USDA class D (animals upon which experiments, teaching, research, surgery, or tests are conducting involving accompanying pain or distress to the animals and for which appropriate anesthetic, analgesic, or tranquilizing drugs are provided). Survival surgery was initiated with ketamine HCl (10–15 mg/kg, IM) followed by butorphanol (0.25 mg/kg, IM). After surgery, all animals were returned to single cages and monitored at 30 minute intervals until they were sitting unaided. Butorphanol was administered for pain relief. Hunched posture, lack of appetite, or different from that typically observed after anesthesia for nonsurgical procedures was interpreted as signs of discomfort or pain. Nonsurgical procedures (including blood and fluid sampling, bone density scans) were conducted using ketamine HCl restraint. Animals were observed from the time of anesthesia until they were fully capable of sitting unaided. Procedures were generally conducted in the A.M. Further monitoring (after the animal was sitting unaided) was done on an hourly basis. Veterinary care was sought if any of the aforementioned signs of distress were observed following surgical or non-surgical procedures.

**Fig 1:**
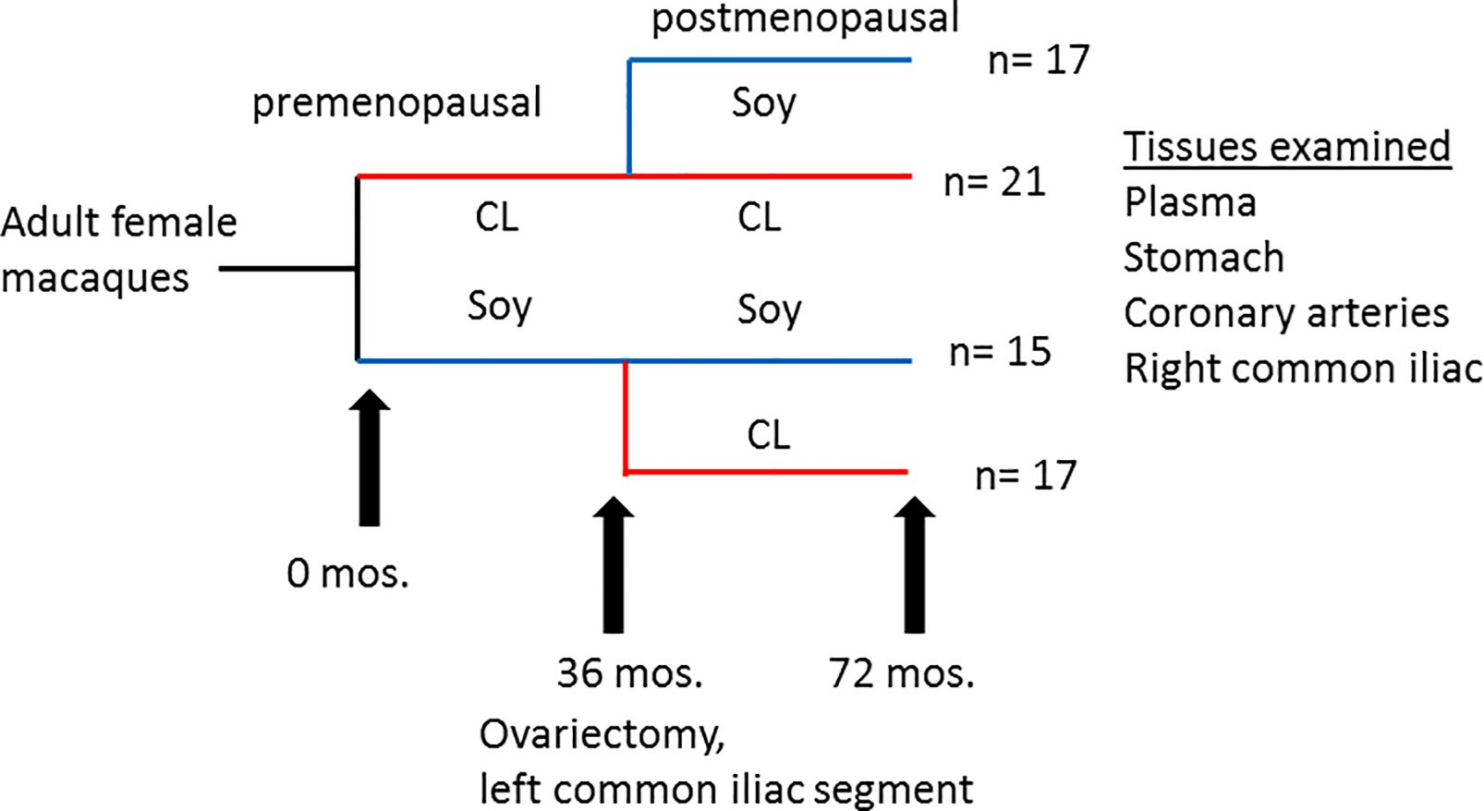
Study design. Cynomolgus macaques received either SOY or CL diets for 36 months, at which point animals were ovariectomized and a left common iliac segment was removed. Half of the animals received the alternate diet for an additional 36 months. The number (n) of surviving animals that could be classified as *H*. *pylori*-positive or–negative (70 out of 74) is given for each group.

A crossover study was conducted as previously described (Fig 1) [38]. Briefly, adult female cynomolgus macaques (Macaca fascicularis) living in permanent social groups (n = 5 or 6 per group x 16 groups; N = 94) were randomized to receive either an atherogenic diet containing casein and lactalbumin (CL) as the protein source or an otherwise identical diet that substituted high-isoflavone soy protein isolate (SOY) for the animal protein. Both diets were formulated to be equivalent in caloric content of protein, fat, carbohydrates and cholesterol (0.28 mg cholesterol/Cal) and to mimic a typical diet consumed by women in the United States [39]. After 36 months, the animals were ovariectomized to induce menopause and a segment of the left common iliac (LCI) artery was removed to measure premenopausal atherosclerosis. Half of the social groups were then randomly selected to receive the alternate diet, while the remaining social groups continued with the original diet. The four resulting diet groups were designated CL-CL (casein/lactalbumin only), CL-SOY, (casein/lactalbumin followed by high isoflavone soy), SOY-CL (soy followed by casein/lactalbumin), or SOY-SOY (soy isolate only). The postmenopausal phase continued for an additional 36 months. Using procedures developed in numerous prior studies [40, 41], animals within each social group were categorized on the basis of competitive interactions as being socially dominant (n = 2 or 3/group) or subordinate (n = 3/group); thus approximately half of the animals were considered dominant and half subordinate.

### Plasma lipid measurement

Total plasma cholesterol (TPC), high-density lipoprotein-associated cholesterol (HDLc), and plasma triglycerides (TGs) were determined on numerous occasions as well as at the end of the study by the Wake Forest Primate Center Clinical Chemistry Laboratory [42, 43], which is fully standardized with the Centers for Disease Control and Prevention-National Institutes of Health Lipid Standardization Program specifications for accuracy and precision. Non-HDLc, which approximates the sum of low-density lipoprotein (LDL) cholesterol and very-low-density lipoprotein cholesterol, was calculated by subtracting HDLc from TPC.

### Screening for viral infections

Serum from each animal was submitted for viral detection. Antibodies against Herpes B virus, simian immunodeficiency virus (SIV), measles, and simian T cell leukemia virus (STLV-1 and -2) were assessed by ELISA. Rhesus cytomegalovirus (RhCMV), simian type D retrovirus (SRV/D), and STLV-1 and-2 were detected by immunofluorescence assay (CNPRC Pathogen Detection Laboratory, Davis, CA).

### Necropsy procedures

At the conclusion of the study, surviving monkeys (n = 74) were euthanized using sodium pentobarbital (100mg/kg, IV; i.e. consistent with the recommendations of the panel on euthanasia of the American Veterinary Medical Association) and perfused with lactated Ringer’s solution. A complete necropsy was performed and all major organs, including the stomach, were collected and fixed in 10% neutral buffered formalin (NBF). The right common iliac artery (RCI) was removed for comparison with the left common iliac artery that was removed at the time of ovariectomy. Hearts were removed and coronary arteries were perfused-fixed with 10% NBF for 1h at 100 mm Hg pressure. The left anterior descending (LAD), left circumflex (LCX), and right coronary arteries (RCA) were then collected. Each artery was sectioned into five blocks (3 mm each in length), cut perpendicular to the long axis of the artery, processed routinely, and embedded in paraffin to generate formalin-fixed paraffin-embedded (FFPE) blocks. Whole sections of the left circumflex artery were also sent to the Uniformed Services University of the Health Sciences for detection of *H*. *pylori*. Due to postmortem degradation, usable gastric tissue could not be obtained from three animals. Coronary artery tissue from these animals was included in the analysis only if *H*. *pylori* was detected at that site, since lack of *H*. *pylori* in the artery does not guarantee that the animal was *H*. *pylori-*negative in the stomach.

### Histopathology and morphometry

For each animal, the first 5mm FFPE block of the LCX (n = 74) was serially sectioned at 5um thickness and sections were mounted on slides. The first five sections were stained with Verhoeff -van Gieson stain to highlight the internal elastic lamina, and plaque size was determined using computer assisted morphometric analysis (ImageProPlus 5.1); the cross-sectional area of plaque (defined as the area between the endothelial lining and internal elastic lamina) in each of the five sections was measured and plaque area for each animal was calculated as the mean of the five sections examined. Section 6 was stained with Hematoxylin-Eosin (H&E), examined by light microscopy for the presence of mineralization and/or necrosis within the tunica intima, and semi-quantitatively scored as follows: 0 = none, 1 = minimal, 2 = mild, 3 = moderate, 4 = marked, 5 = severe. Section seven was stained with Masson’s Trichrome to highlight areas of collagen deposition (fibrosis). The proportion of intima occupied by fibrosis was determined by overlay of a 25 x 25 pixel grid with evaluation of each crosshatch as positive or negative for collagen by a board-certified pathologist (JAC) blinded to treatment. The remaining LCX serial sections were utilized for immunohistochemistry as detailed below. Additional 5 μm sections of stomach tissue and LCX artery were generated, mounted on slides and stained with H&E and Genta-Robason (H&E, silver stain and Alcian blue), which is a conventional method for detecting the presence of *H*. *pylori*. Slides were examined by light microscopy for the presence/absence of argyrophilic curved or spiral-shaped bacteria. The remaining slides were used for *in situ* hybridization as detailed below.

### Histochemistry and immunohistochemistry

For immunohistochemistry, the slides were incubated with mouse anti-human HAM56 (Dako Cytomation, Carpinteria, CA), mouse anti-human CD3 (BDBiosciences, San Jose, CA), mouse anti-human CD4 (Abcam, Cambridge, MA), and mouse anti-human CD8 (Abcam, Cambridge, MA) for 1.5 h at 25° C. Primary antibodies were localized with appropriate biotinylated secondary antibodies, streptavidin–alkaline phosphatase (Biogenex, San Ramon, CA) and Vector Red (Vector Labs, Burlingame, CA) substrate. Sections were counterstained with Mayer's Hematoxylin and examined by the study pathologist (JAC) using light microscopy. Thymus and lymph node were used as tissue controls; as an assay control, the primary antibodies were substituted with non-immune sera. Cell densities for macrophages, CD3+ cells, CD4+ cells, and CD8+ cells were determined by overlay of a 25 x 25 pixel grid with evaluation of each crosshatch for positive or negative staining by the pathologist (JAC), who was blinded to treatment. Cell densities are expressed as the percentage of intima occupied by positive staining.

### RNA isolation and quantification

RNA was isolated from intima-media sections as described previously [44–46] using a Tri-Reagent protocol. Total RNA concentration and quality was assessed using an Agilent 2100 Bioanalyzer and aliquots were reverse transcribed using a high-capacity cDNA archive kit (Applied Biosystems). Quantitative real-time reverse transcriptase–polymerase chain reaction (PCR) was performed on an ABI Prism 7000 system using TaqMan FAM-MGB primer-probe assays (Applied Biosystems). Primers included cynomolgus macaque-specific primers (CCL2, encoding monocyte chemotactic protein-1 [MCP-1], VCAM1 [vascular cellular adhesion molecule-1], ICAM1 [intercellular adhesion molecule-1], CD3G, encoding the CD3 δ chain, CD4, ESR1, encoding estrogen receptor alpha [ER-α], and ESR2, encoding estrogen receptor beta [ER-β]) and human-specific primers (microsialin/CD68, T-cell receptor-β, and IL6 [interleukin-6]). Quantitative reverse transcriptase–PCR data were normalized to the geometric mean of the endogenous, constitutively expressed control genes glyceraldehyde-3-phosphate dehydrogenase, β-actin, and ribosomal protein L13a [47] using the 2^–ΔCT^ procedure [48, 49]. Amplifications not reaching threshold by 40 cycles were designated as zero expression.

### *In situ* hybridization

*H*. *pylori* was detected in coronary arteries (i.e., the LCX) and stomach tissue following euthanasia. All procedures were performed on paraffin sections according to previously described methodologies [Liu, 18648543; [50, 51]]. *H*. *pylori-*16S rRNA (antisense 5’-TAC CTC TCC CAC ACT CTA GAA TAG TAG TTT CAA ATGC-3’ and sense 5’ CTA TGA CGG GTA TCC GGC-3’) and *H pylori cagA* (antisense 5’CTG CAA AAG ATT GTT TGG CAG A-3’ and sense 5’-GAT AAC AGG CAA GCT TTT GAG G-3’) probes were used. The 5' ends of the oligonucleotides were labeled with biotin or digoxygenin (see below). All probes were synthesized in the Synthesis and Sequencing Facility, Biomedical Instrumentation Center (USUHS). Negative controls were routinely run as described previously [52]. These included using sense instead of antisense probes, hybridization buffer without probe, probe without biotin or digoxigenin label, pre-treatments with DNase I, RNase A or both, and incubation with an irrelevant probe, scorpion Buthus martensi Karsch neurotoxin (5’-GGC CAC GCG TCG ACT AGT AC-3’)[53].

*In situ* hybridization was performed as previously described [52, 54, 55]. Briefly, sections were deparaffinized in xylene, rehydrated, treated with proteinase K and washed with standard saline citrate solution (SSC). For pre-hybridization, sections were incubated with hybridization mixture (Denhardt’s solution, NaCl, formamide, dextran sulfate EDTA, bovine serum albumin) and hybridization solution (SSC and extracted salmon testis DNA). For hybridization, sections were incubated with the denatured primer overnight at 37˚C. For post-hybridization, unbound probe was removed by successive washes in decreasing concentrations of SSC. For visible light studies, biotin-labeled probes were detected using streptavidin-alkaline phosphatase and nitro blue tetrazolium/5-bromo-4-chloro-3-indolyl-phosphate (NBT/BCIP). Sections were counterstained with Nuclear Fast red. Serial sections were used to detect 16S rRNA and *cagA* mRNA. For fluorescence dual detection studies, 16S rRNA probes were labeled with biotin and detected using avidin-Texas red (red color), while *cagA* probes were labeled with digoxigenin-3-0-methylcarbonyl-ε-aminocaproic acid-N-hydroxy-succinimide ester (DIG-NHS ester, Roche Diagnostics, Indianapolis, IN) and detected using anti-digoxigenin-FITC (green color).

An Eclipse Nikon microscope was used to examine the section at X100, X400 and X1000 magnification for bright light and for fluorescence. Bacterial density was quantified as described previously [52]. Briefly, the number of positive-stained bacteria were quantified at 400X magnification in three randomly-selected fields according to a modification of the point-counting stereological method and using an intraocular reticle (27 mm diameter, covering 3,578 μm^2^, i.e. 17,892 μm^3^ for 5 μm-thick sections; No. KR-409 (Klarmann Rulings, Inc. Litchfield, NH), located in a Nikon Eclipse E 800. Tissues were analyzed randomly and blindly regardless of category. All data were expressed as mean ± standard error of the mean (SEM) number of bacteria per 10^6^ μm^3^ (representing an imaginary cube with sides of 100 μm or 0.1 mm).

### Statistical analysis

Descriptive statistics and graphical plots, such as means, standard deviations and standard errors, and comparative boxplots, were calculated and obtained for *H*. *pylori* densities, *cagA* densities, *H*. *pylori* infection status, and social status. Linear models and analysis of variance (ANOVA) were used to determine the influences of *H*. *pylori* infection, diet, and social status on severity of premenopausal and postmenopausal atherosclerosis, arterial inflammatory markers, and lipid profiles. Pairwise group comparisons were performed using Welch two-sample t-test and/or nonparametric rank tests (Kruskal-Wallis H test), the latter employed when assumptions for the validity of the t-test were not satisfied. Linear models and correlation analyses were used to compare the effect of *H*. *pylori* density in the artery and stomach on plaque area and thickness. Fisher’s exact test was used where indicated. The (effective) sample sizes in some of the calculations were smaller than the overall sample sizes due to missing values.

## Results

### Evidence of *H*. *pylori* infection in gastric and artery tissue

**Fig 2:**
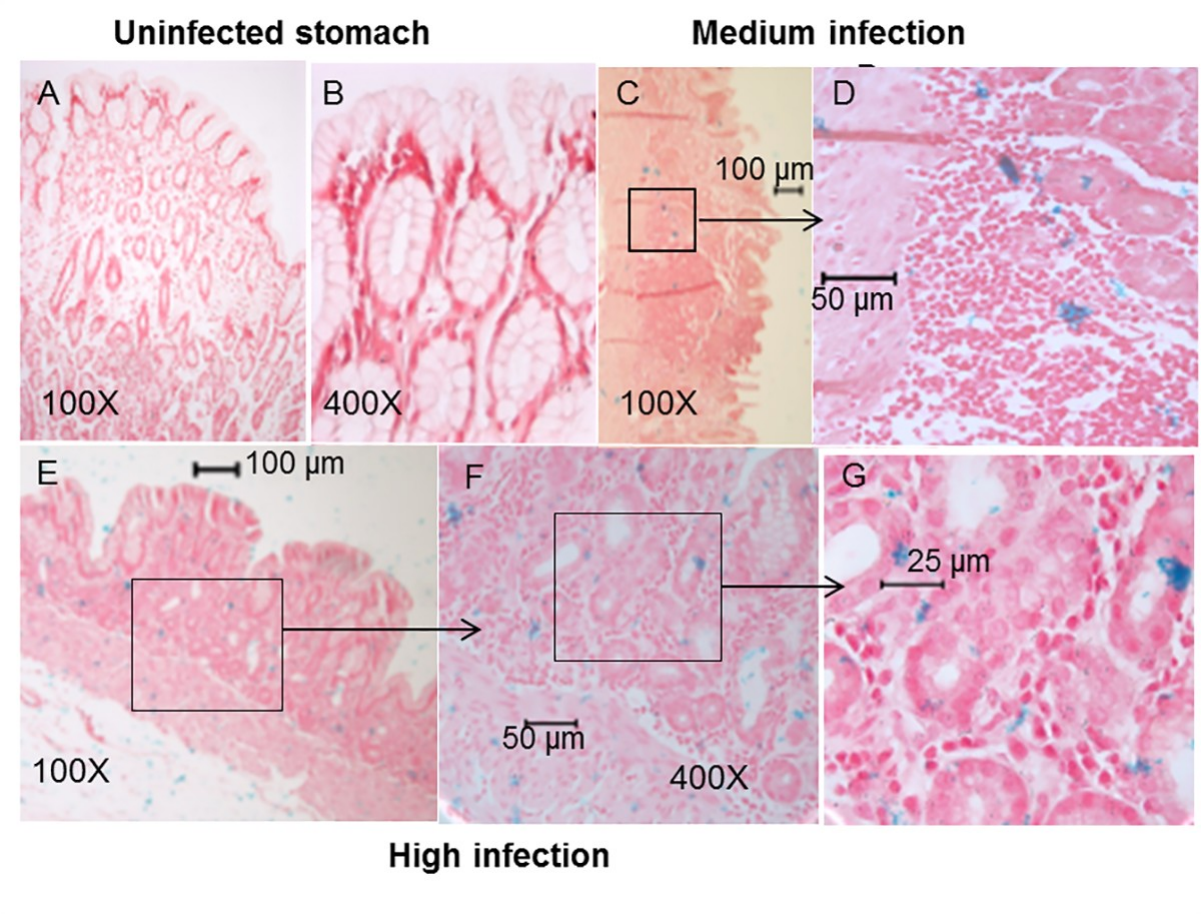
Detection of *H*. *pylori* in gastric tissue. *In situ* hybridization using the *H*. *pylori-*specific 16S rRNA probe (blue staining) demonstrates absence and presence of *H*. *pylori* in gastric sections. Sections were counterstained with Nuclear Fast Red. A, B) Stomach from an uninfected animal (no blue staining). C, D) Stomach showing a medium infection density. E, F) Stomach showing a high density of *H*. *pylori*.

**Fig 3:**
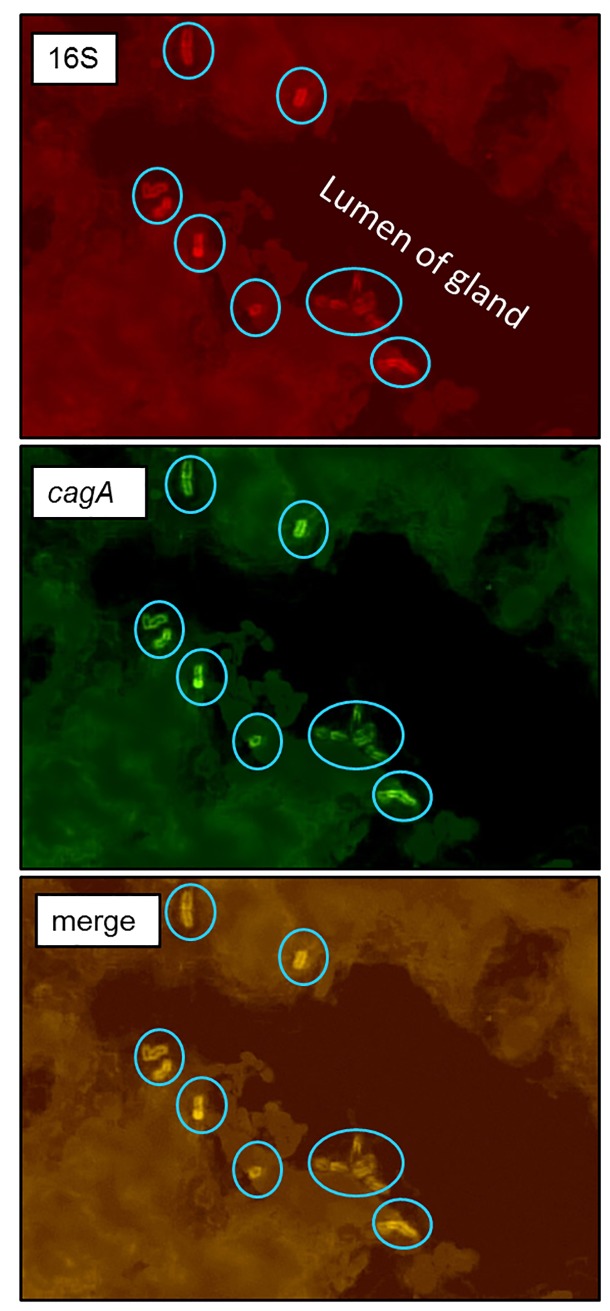
Identification of *cagA-*positive *H*. *pylori* in gastric tissue. Fluorescence *in situ* hybridization of *H*. *pylori* 16S rRNA (red) and *cagA* (green) was done on tissue sections. *H*. *pylori* (circled) are seen attached to epithelial cells within a gastric gland. Colocalization of red and green staining indicates that the organisms are *cagA-positive*.

The study design is outlined in Fig 1. Curved or spiral organisms consistent with *H*. *pylori* were first detected in gastric mucosa using the Genta-Robason staining procedure. Subsequently live *H*. *pylori* were specifically detected in gastric mucosa and LCX coronary artery sections by *in situ* hybridization using RNA anti-sense probes specific for *H*. *pylori* 16S rRNA or *cagA* mRNA. These probes recognize RNA and the method has been previously validated [52]. The normal infection pattern for *H*. *pylori* is for the majority of organisms to reside in the mucus just above the epithelium, attached to epithelial cells, or within gastric crypts. *H*. *pylori* is occasionally intracellular or found within gastric capillaries[56]. We found similar results in our study. Within the stomach, the majority of *H*. *pylori* were located in the superficial gastric mucosa (Fig 2), but a few organisms were located in gastric blood vessels. *In situ* hybridization demonstrates co-expression of 16S rRNA and *cagA* in the same bacteria (Fig 3), further confirming that the organisms are *H*. *pylori* and demonstrating that some macaques were infected with *cagA*-positive strains, some were infected with *cagA-*negative strains, and some were infected with a mixture of strains. Infection with multiple *H*. *pylori* strains is not unusual.

**Fig 4:**
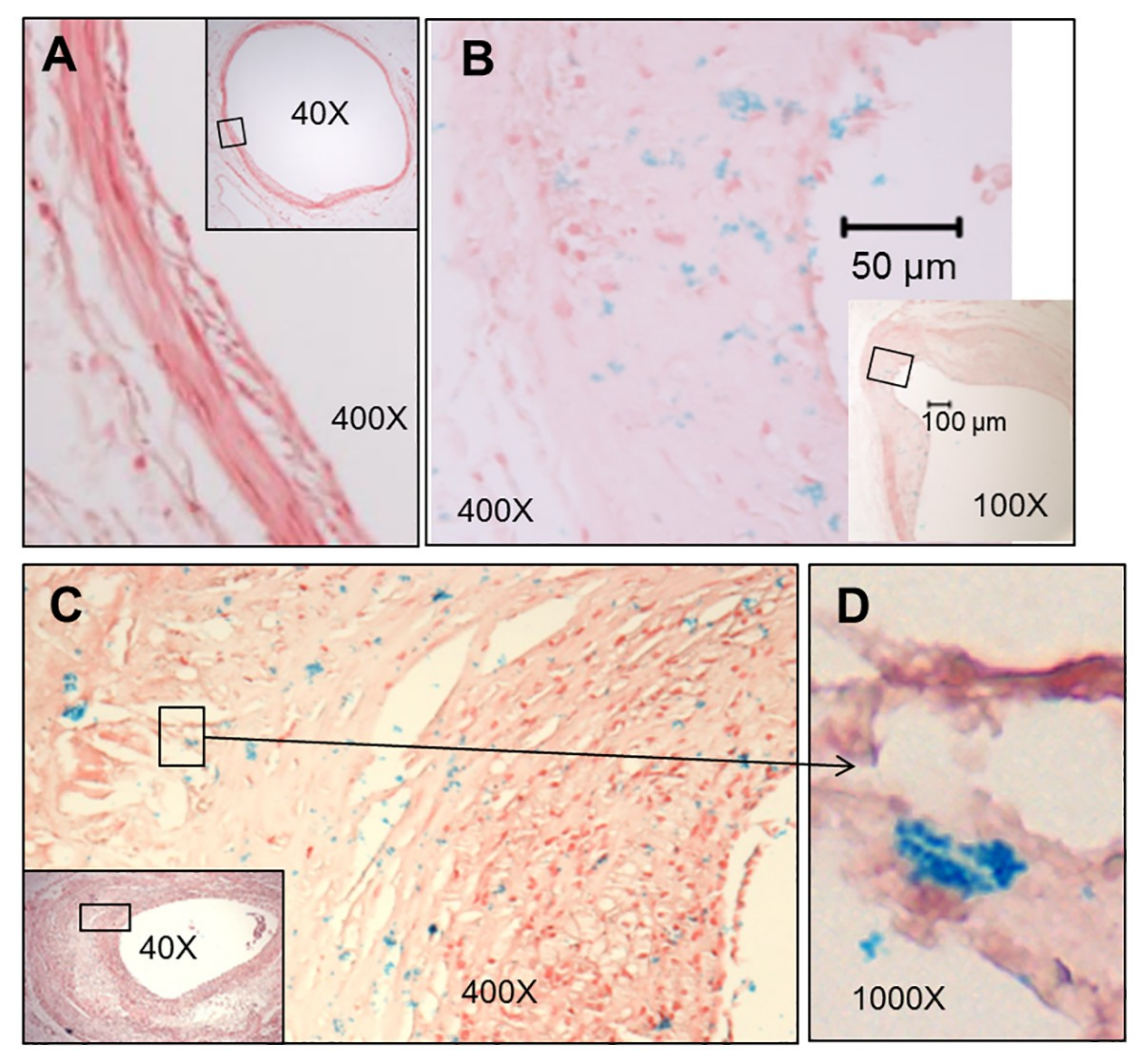
*In situ* hybridization using the *H*. *pylori* 16S rRNA probe (blue staining) demonstrating presence and absence of *H*. *pylori* in left circumflex artery sections. Sections were counterstained with Nuclear Fast Red and boxed regions of low magnification images indicate the locations of the high magnification images. A) Artery from an uninfected animal. B) Artery showing a medium infection density (blue dots). C) Artery showing a high density of bacteria. D) High magnification reveals a cluster of *H*. *pylori* suggestive of limited growth.

**Fig 5:**
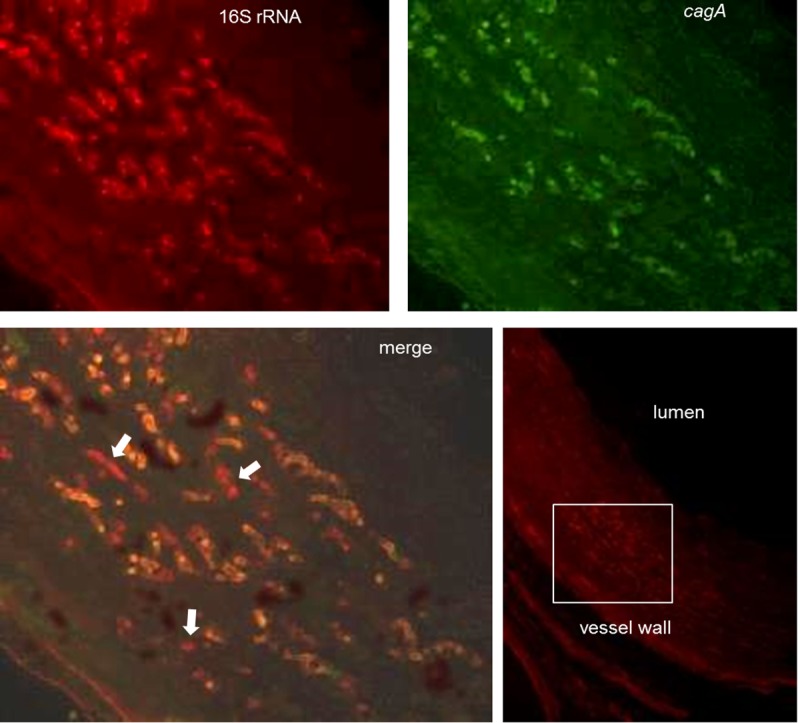
Fluorescence *in situ* hybridization on an artery section demonstrating mixed *cagA*-positive and *cagA*-negative *H*. *pylori*. Fluorescence *in situ* hybridization of *H*. *pylori* 16S rRNA (red) and *cagA* (green) was conducted on tissue sections and imaged using confocal microscopy. White arrows indicate *H*. *pylori* lacking *cagA* expression. The bottom right panel shows the region of interest (white box) in a lower magnification image.

**Fig 6:**
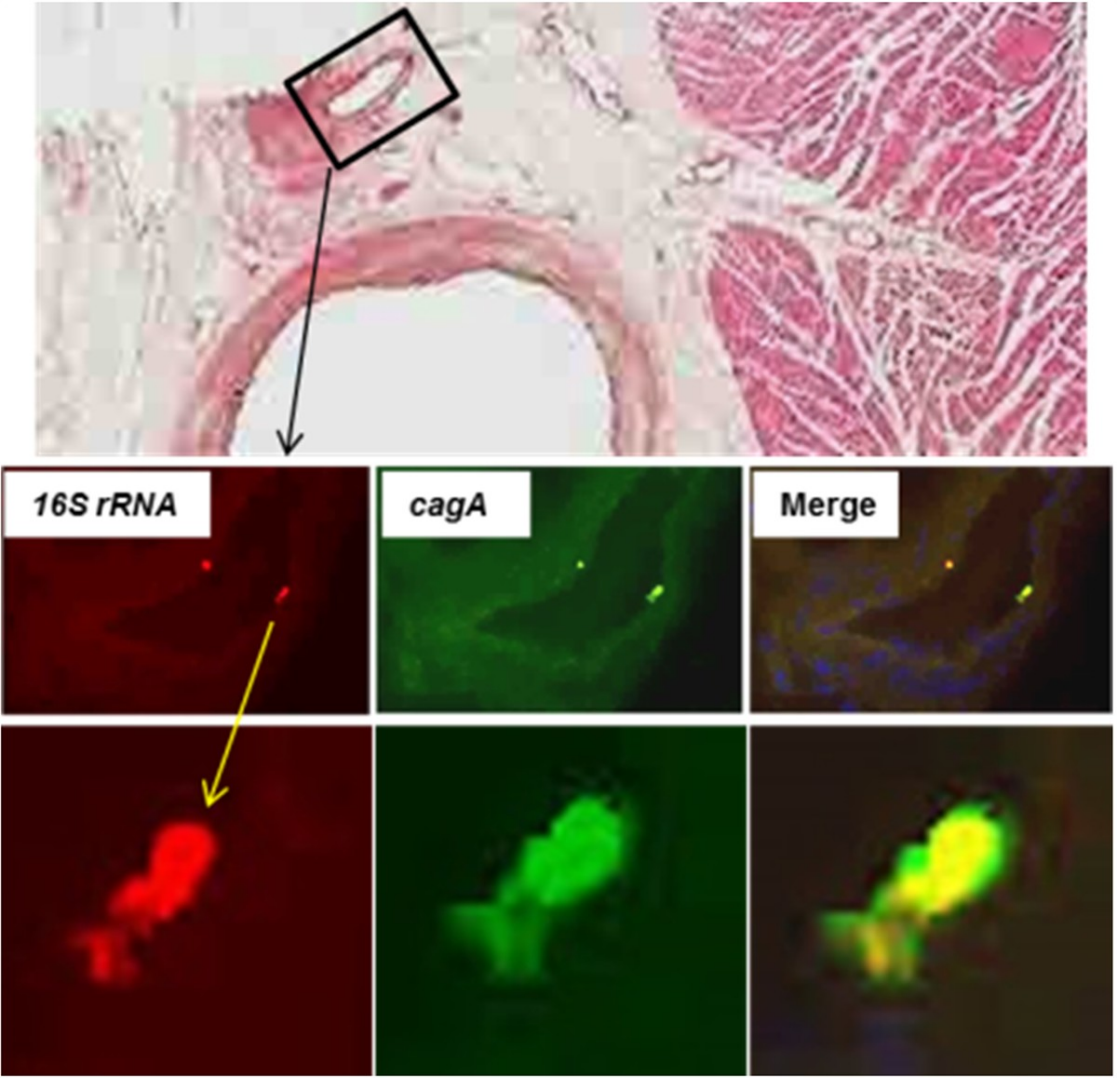
*H*. *pylori* attached to the endothelium of a collateral arteriole. Fluorescence *in situ* hybridization of *H*. *pylori* 16S rRNA (red) and *cagA* (green) was carried out on tissue sections with DAPI staining of nuclei (blue). Top image, boxed area shows the arteriole shown in the lower images. Middle row, 400x; Bottom row, 1,000x.

**Fig 7:**
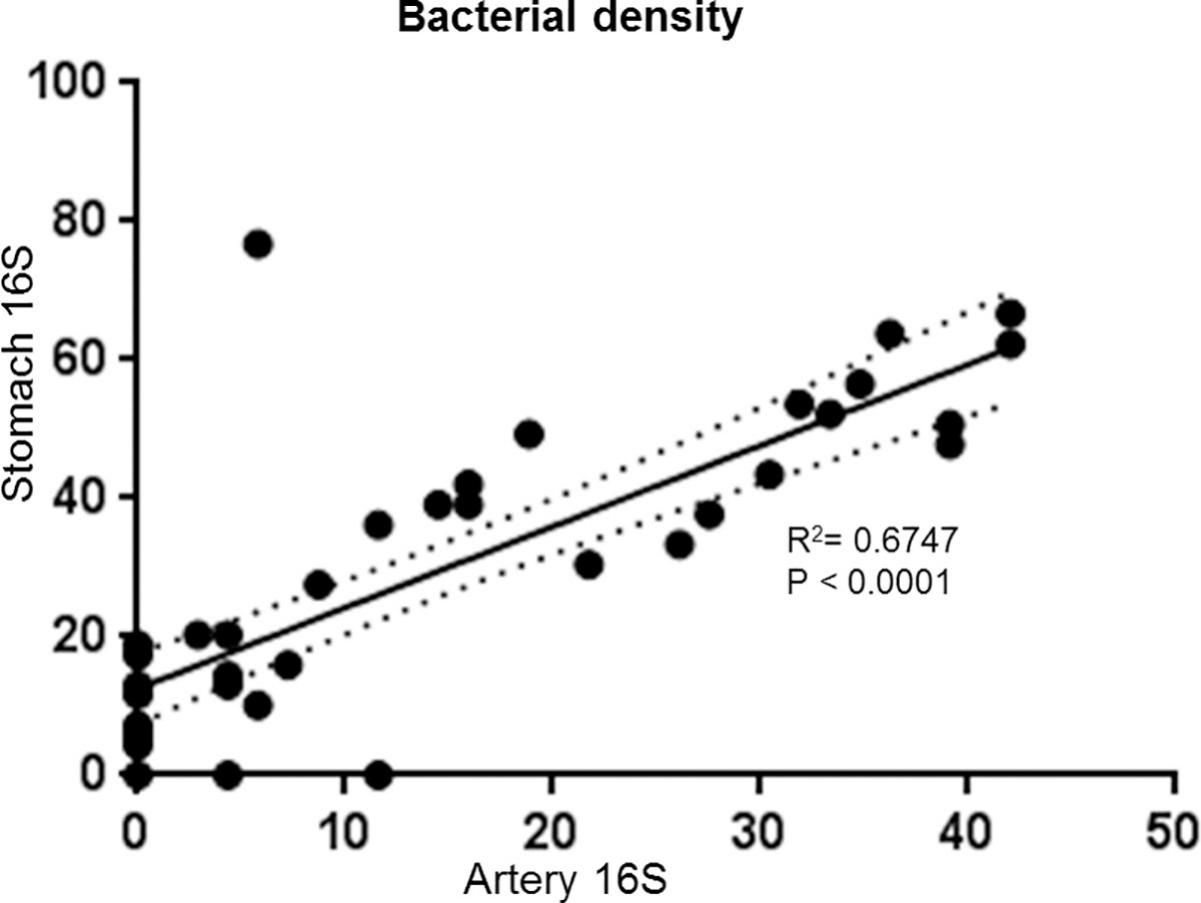
The density of *H*. *pylori* in the stomach correlates with the density in the left circumflex coronary artery. *H*. *pylori* density was determined by counting the number of bacteria per field (as determined by 16S rRNA *in situ* hybridization) and is expressed as bacteria per 10^6^ μm^3^. Only *H*. *pylori* positive animals with both stomach and artery data are included in the analysis (n = 44). Solid line, linear regression best fit; dotted line, 95% confidence interval; R^2^, coefficient of determination.

Of potentially greater interest, *H*. *pylori* was detected within coronary atherosclerotic plaques as isolated organisms, or small colonies, randomly distributed throughout the fibrofatty admixture of foam cells and extracellular matrix. Fig 4 shows examples of uninfected, moderately infected, and highly infected LCX arteries as measured using colorimetric ISH with a 16S probe. Tunica intima and media were often involved, but no colonization was observed in the tunica adventitia, suggesting that *H*. *pylori* originates from the vessel lumen and does not easily cross the external elastic membrane. Bacteria often appeared in clusters, suggesting limited replication (Fig 4D). Using serial sections, some *H*. *pylori* were found to express *cagA*. This latter observation was confirmed by dual fluorescence in which 16S rRNA was stained with Texas Red and *cagA* mRNA was stained with FITC (green) (Fig 5). Occasional individual bacteria were noted in collateral vessels (Fig 6), but there was no evidence of bacterial growth in non-atherosclerotic vessels. Animals were defined as *H*. *pylori* positive if *H*. *pylori* was visualized in either gastric or coronary artery sections following euthanasia. Iliac arteries were not assessed for *H*. *pylori* infection. Of the animals with evaluable data sets, 64% were *H*. *pylori-*positive. Thus, there were adequate numbers of infected and uninfected animals for statistical determination of the effects of *H*. *pylori* infection on atherosclerosis, arterial inflammation, and plasma lipids. All but seven of the 45 *H*. *pylori-*positive animals were infected with at least some *cagA-*positive strains, with the percentages of *cagA-*positive bacteria varying between 11% and 100% in a single artery section. Four animals were infected with only *cagA-*positive strains, while the remainder had mixed infections with both *cagA-*positive and *cagA-*negative strains, as seen in Fig 5. Of the *H*. *pylori-*positive animals, 64% had evidence of *H*. *pylori* infection in both the stomach and arteries and 29% had *H*. *pylori* only in the stomach. As expected, the density of *H*. *pylori* was highest in the gastric mucosa, but impressive numbers of *H*. *pylori* were found in the LCX arteries (up to 43 per 10^6^ μm^3^). When the densities of *H*. *pylori* in the coronary arteries were plotted against gastric mucosal densities, a substantial linear correlation (coefficient of determination of R^2^ = 0.6747, p< 0.0001) was found (Fig 7). A correlation matrix of *H*. *pylori* 16S and *cagA* densities in the stomach and LCX coronary artery revealed correlations of r ≥0.73 for all pairings, further indicating that the densities of *cagA-*positive or–negative *H*. *pylori* in the artery are significantly associated (p< 0.0001) with densities in the stomach. In other words, live *H*. *pylori* were frequently present in coronary arteries of *H*. *pylori-*infected monkeys and the density of bacteria found in plaques was strongly related to colonization density in the stomach.

### *H*. *pylori* infection influences severity of premenopausal and postmenopausal atherosclerosis

The influence of *H*. *pylori* infection (as determined in postmenopausal gastric/coronary tissue) was evident at both premenopausal and postmenopausal time points. Premenopausal atherosclerosis was assessed in the LCI at the time of ovariectomy and postmenopausal atherosclerosis was assessed in both the RCI and coronary arteries (LCX, LAD, RCA). Both the intimal area (IA) and maximum intimal thickness (MXIT) were strongly associated with *H*. *pylori* infection (p<0.005) even when diet was included in the statistical model (Table 1). Few *H*. *pylori-*negative animals had plaques of appreciable size at the premenopausal time point. Only 16% of *H*. *pylori-*negative animals had intimal areas greater than 0.5 mm^2^, whereas 55% of infected animals had premenopausal plaque areas above that value (p< 0.001 by Fisher’s exact test). There was no significant association between *H*. *pylori* infection status and premenopausal estrogen receptor expression. Other premenopausal measurements shown in Table 1 will be discussed below.

**Table 1:** Premenopausal iliac artery plaque parameters and inflammatory biomarkers.

	*H*. *pylori* negative		*H*. *pylori* positive		Ratio of Means	
	Mean (n)^b^	SEM	Mean (n)	SEM	Hp+/Hp-	*P-* Value^a^^,^ ^c^
Max plaque thickness	0.1451 (25)	0.0223	0.2555 (45)	0.0207	1.7607	**0.0003** **
Plaque area	0.2419 (25)	0.0566	0.5361 (45)	0.0614	2.2165	**0.0004** **
Mineralization	1.4000 (25)	0.3416	2.1591 (44)	0.2640	1.5422	**0.0423** *
Intimal collagen	28.2063 (25)	5.7756	41.6703 (42)	3.1962	1.4773	**0.0241** *
Intimal HAM56+	0.6943 (25)	0.3766	2.0685 (44)	0.3622	2.9791	**0.0054** **
CD68	0.0522 (23)	0.0192	0.1332 (41)	0.0224	2.5501	**0.0040** **
CD3	0.0017 (23)	0.0004	0.0031 (41)	0.0005	1.7664	**0.0206** *
CD4	0.0135 (23)	0.0032	0.0269 (41)	0.0046	1.9907	**0.0104** *
MCP-1	0.0126 (23)	0.0040	0.0219 (41)	0.0039	1.7387	0.0542ns
VCAM-1	.0000355 (23)	.000000782	.00000588 (41)	.00000131	1.6526	0.0661ns
ICAM-1	0.0080 (23)	0.0014	0.0127 (41)	0.0014	1.5923	**0.0099** **
ER-α	0.0085 (23)	0.0006	0.0074 (41)	0.0005	0.8723	0.1875 ns (2-sided)
ER-β	0.000125 (23)	0.0000177	0.0000932 (41)	0.00000786	0.7438	0.1079ns (2-sided)
IL-6	0.000442 (23)	0.0000934	0.000864 (41)	0.000152	1.9531	**0.0108** *

^a^Bold text indicates statistical significance. Unless otherwise indicated, the p-value are based on a one-sided Welch Two-Sample T-Test with alternative hypothesis that the mean for the *H*. *pylori* negative is smaller than the mean for the *H*. *pylori* positive.

^b^(n) represents the effective sample size used in computing the mean and standard error of the mean (SEM).

^c^[ns = not significant

* = significant at 5% level

** = significant at 1% level.]

**Fig 8:**
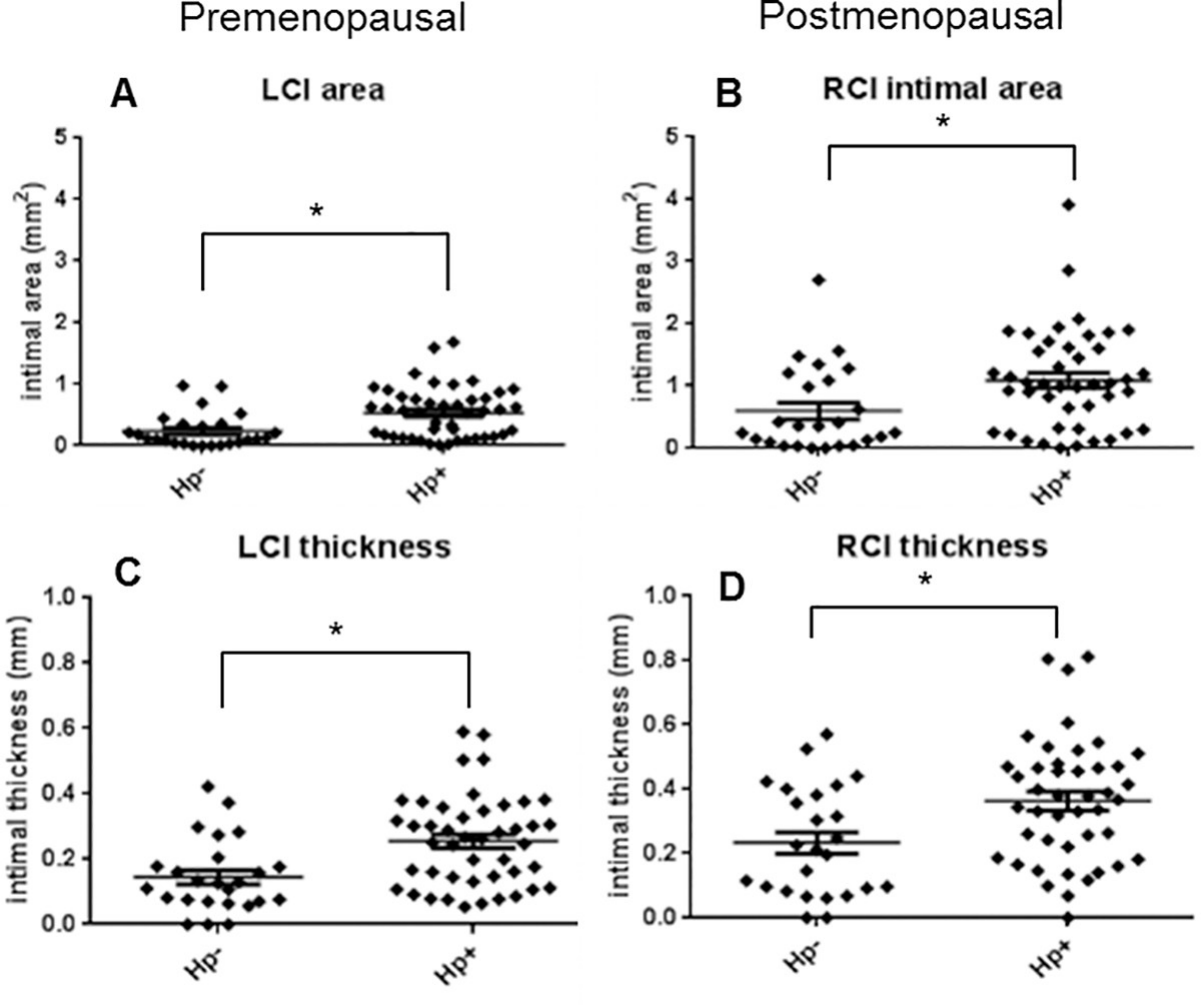
Premenopausal and postmenopausal effects of *H*. *pylori* on atherosclerotic plaque thickness. Common iliac artery plaque intimal areas and maximum intimal thicknesses were measured at premenopausal (A and C, n = 70) and postmenopausal time points (B and D, n = 69). Hp, *H*. *pylori;* LCI, left common iliac; RCI, right common iliac). Graphs show mean +/- standard error of the mean. *P< 0.05.

As shown in Fig 1, following ovariectomy and for an additional 36 months, animals were either fed the same diet they had consumed premenopausally or switched to the other diet. MXIT and IA measurements were taken on the right common iliac artery and three coronary arteries (LCX, LAD, and RCA). Postmenopausal results are summarized in Table 2. Fig 8B and 8D show measurements of iliac artery plaques at the postmenopausal time point as compared with premenopausal values. Progression of iliac plaque area, as determined by subtracting premenopausal MXIT and IA from postmenopausal values in each animal, trended higher in *H*. *pylori-*infected animals, but progression of plaque thickness was unaffected by infection status.

**Table 2:** Postmenopausal plaque measurements and lipid profiles.

	H. pylori negative		H. pylori positive		ratio	
	mean	SEM	mean	SEM	Hp+/Hp-	p value
avg. coronary area	0.331	0.076	0.961	0.236	2.90	**0.0070**
avg. coronary thickness	0.121	0.021	0.282	0.042	2.32	**0.0006**
iliac area	0.601	0.137	1.090	0.122	1.82	**0.0048**
iliac area progression	0.359	0.097	0.545	0.083	1.52	0.0750
iliac thickness	0.233	0.034	0.364	0.029	1.56	**0.0024**
iliac thickness progression	0.088	0.022	0.105	0.020	1.19	0.2898
total cholesterol	217.52	13.54	242.16	12.01	1.11	0.0894
triglycerides	34.52	3.95	35.39	6.47	1.03	0.4547
HDL cholesterol	66.63	4.84	49.57	2.92	0.74	**0.0048 (2-sided)**
LDL + VLDL cholesterol	151.00	16.19	192.70	13.21	1.28	**0.0255**
TPC/HDLc	3.89	0.50	5.75	0.44	1.48	**0.0036**

Bold text indicates statistical significance. There were 25 observations for *H*. *pylori*-negative group and 44 observations for *H*. *pylori* -positive group. P-values are obtained from Welch two-sample t-test based on a one-sided alternative hypothesis (except for HDL cholesterol, which is two-sided).

**Fig 9:**
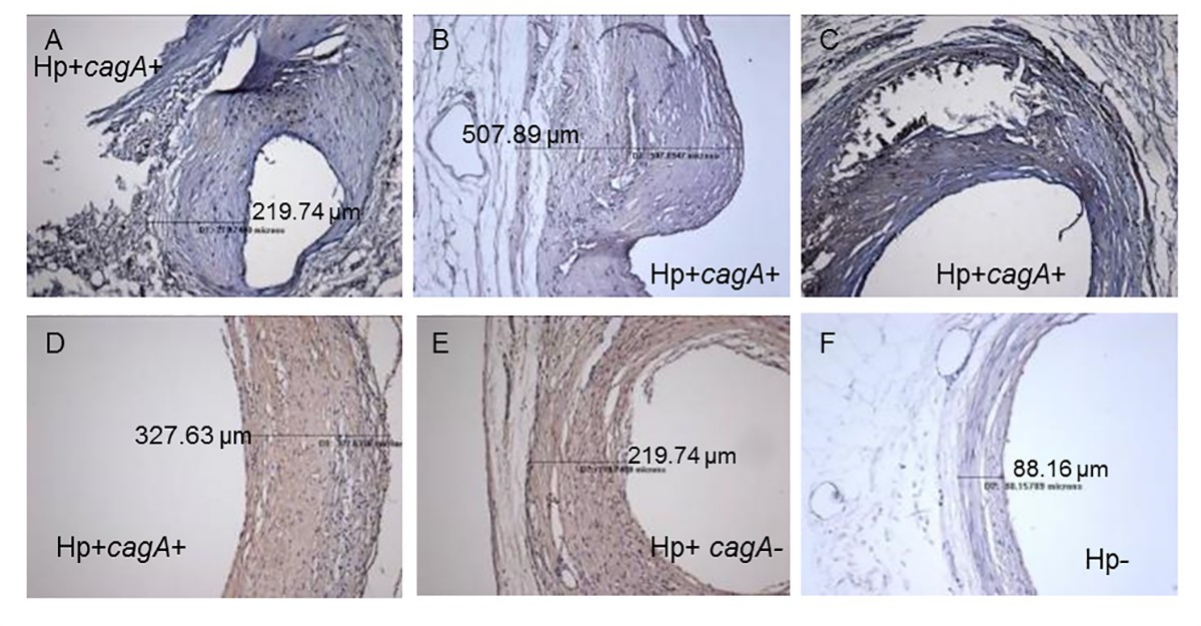
Coronary artery pathology seen in *H*. *pylori-*infected and uninfected macaques. Panels A, B, C, and D show arteries from *cagA* positive (*cagA+*) animals. Panels A and C show severe necrosis. Panel B shows an atypical bulging plaque. Panels D, E, and F depict arteries that are near the median intimal thickness values for *cagA-*positive, *cagA-*negative (*cagA-*), and uninfected (Hp-) animals, respectively. Measurement lines shown in some images indicate the maximal intimal thickness for those sections.

**Fig 10:**
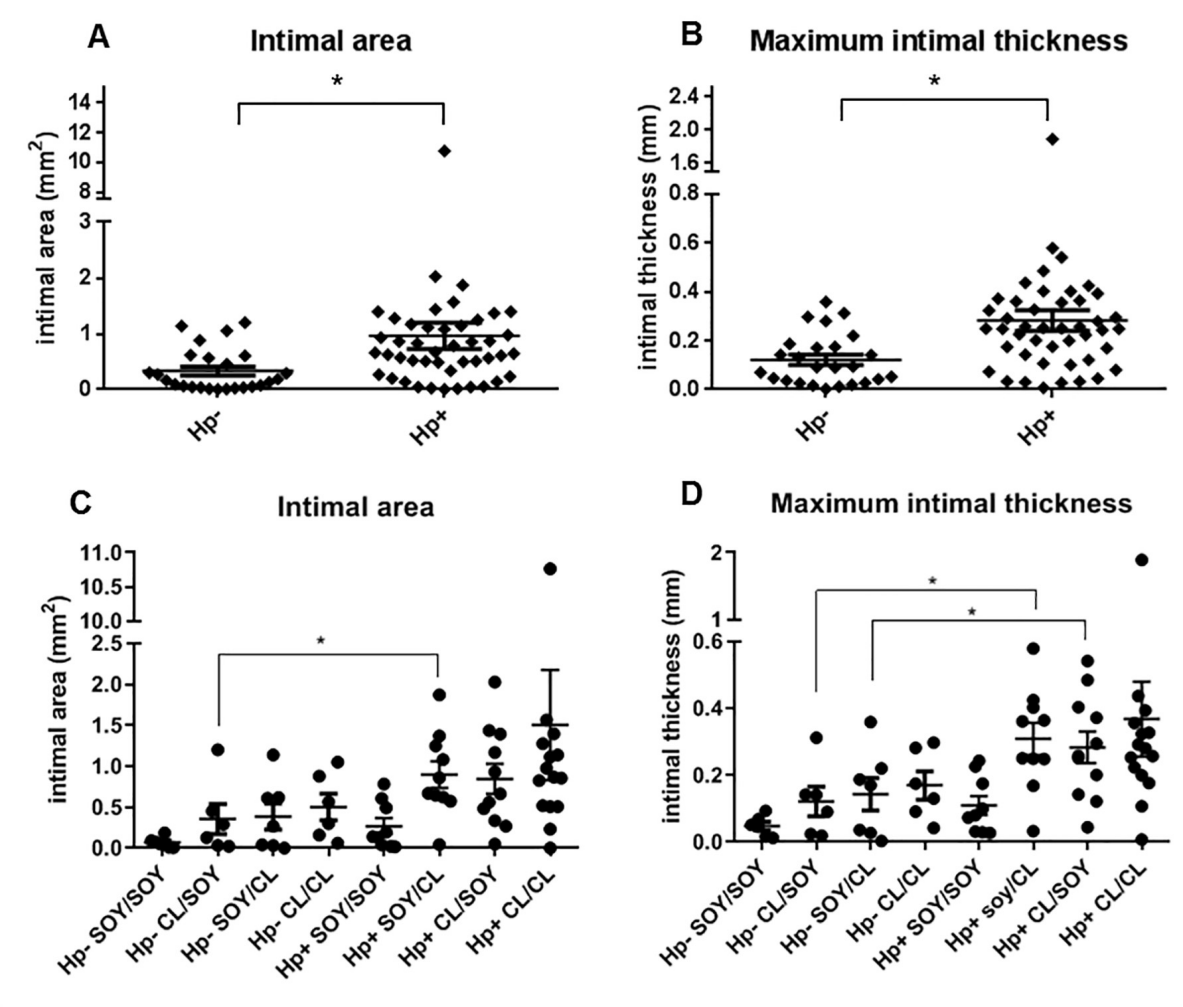
Effects of *H*. *pylori* infection and diet on coronary artery plaque measurements. The values shown represent the average plaque measurements of the left circumflex, left anterior descending, and right coronary arteries. Values are grouped according to *H*. *pylori* (Hp) status (A and B) or according to *H*. *pylori* status and pre/postmenopausal diet group (C and D). Graphs show mean +/- standard error of the mean. *P< 0.05.

The micrographs in Fig 9A–9C represent examples of extreme LCX artery pathology, which was seen in animals infected with *cagA-*positive *H*. *pylori*. Large necrotic regions and bulging plaques in the LCX were found in six of the *cagA-*positive animals and none of the *cagA-*negative or uninfected animals (Fisher’s exact test p< 0.05). Plaques representative of median thickness values for each *cagA-*positive, *cagA-*negative, and uninfected macaques are shown in Fig 9D–9F. Note that these values represent the maximum thickness of the LCX slice in which *H*. *pylori* density was determined, not the maximum thickness of the entire artery section. Plaque measurements for the three coronary arteries were averaged and the differences between *H*. *pylori-*positive and–negative animals irrespective of diet group are shown in Fig 10A and 10B. *H*. *pylori* infection substantially increased intimal area and thickness (p = 0.0004 and p = 0.0003, respectively). Panels 10C and 10D demonstrate the effects of both *H*. *pylori* infection and diet group. *H*. *pylori-*negative animals fed a SOY-SOY diet had essentially no plaque accumulation, whereas several *H*. *pylori-*positive animals on the same diet had modest amounts of plaque. Almost all *H*. *pylori-*positive animals that received a casein/lactalbumin diet at any point had moderate to severe atherosclerosis. Analyses of the effects of diet on atherosclerosis in these animals have been previously published [38, 44], and therefore are only briefly mentioned in this report.

The outlier seen in Fig 10 represents a *cagA-*positive animal receiving the casein-lactalbumin diet for the duration of the study. There is no a priori basis for invalidating the measurements from this animal. Moreover, the results of analyses were similar whether the outlier was included or not, and all statistical analyses discussed herein are inclusive of all animals.

### Effects of *H*. *pylori* on plaque size are due to local rather than systemic effects

We next sought to determine whether the effects of *H*. *pylori* on plaque measurements were due to local effects of bacteria within the plaques or due to systemic effects stemming from gastric infection. To this end, we performed multiple regression analysis and correlation analysis. As shown in Table 3, the density of *cagA-*positive *H*. *pylori* in the LCX coronary artery showed a stronger correlation to plaque area and thickness than did the density of *cagA-*positive bacteria in the stomach. On the other hand, the overall densities of *H*. *pylori*, as measured by 16S in the stomach or artery, did not correlate with plaque measurements (Table 3). Taken together, these data suggest that the presence of *cagA* is critical for artery pathology and that bacteria are acting locally rather than systemically.

**Table 3:** Correlations between plaque measurements and *H*. *pylori* density in the artery or stomach.

	artery 16S		artery cagA		stomach 16S		stomach cagA	
	P value	Corr.	P value	Corr.	P value	Corr.	P value	Corr.
premenopausal iliac plaque area	.0698	.2165	**.0046**	.3329	.1683	.1678	**.0446**	0.2426
premenopausal iliac plaque thickness	.0710	.2156	**.0068**	.3182	.18	.1633	.1015	0.1988
LCX area	0.5241	0.0769	0.0532	.2305	.2427	0.1425	.0595	0.2281
avg. cardiac plaque area	.1043	.3866	**.0268**	.2628	.1474	0.1763	**0.0245**	0.2707
LCX plaque thickness	.2746	.1314	**.0101**	.3034	.1564	0.1725	**0.0263**	0.2674
avg. cardiac plaque thickness	.1930	.1563	**.0061**	.3222	.0877	0.2071	**0.0127**	0.2985
HDL cholesterol	**.0178**	-0.2826	0.0851	-0.2069	**0.0032**	-0.3524	**0.0079**	-0.3197
LD+VLD cholesterol	0.6492	0.0553	0.5853	0.0663	0.2939	0.1292	0.2963	0.1285
TPC/HDLc	0.1338	0.1810	0.2077	0.1524	**0.0400**	0.2497	**0.0466**	0.2422

Bold text indicates statistical significance. Estimates of the Pearson’s correlation coefficient together with their p-values for testing, based on a t-test, that the correlation coefficient is not equal to zero between bacterial densities in the artery or stomach and plaque or plasma lipid measurements.

### *H*. *pylori* infection influences arterial inflammatory markers

**Fig 11:**
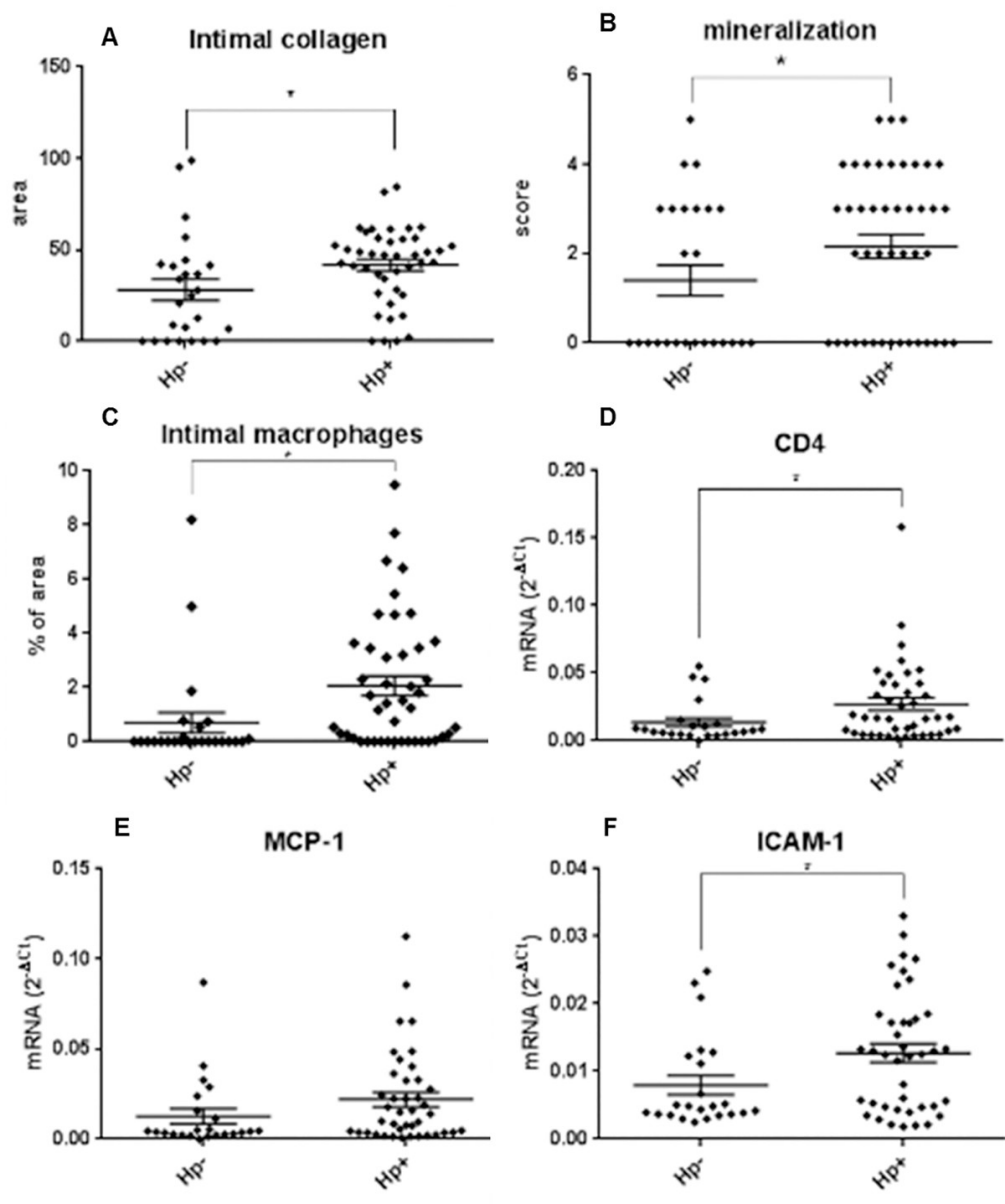
Effect of *H*. *pylori* infection on molecular markers of plaque inflammation. Intimal collagen area was determined by computer-assisted image analysis (A, C). Quantitative PCR was carried out using mRNA from intima-media sections of the left common iliac artery (D-F). Data were normalized to the geometric mean of endogenous control genes GAPDH, ACTB, and RPL0. *p< 0.05. (one-sided two-sample T-test) A, n = 67; B and C, n = 69; D-F, n = 64.

Additional measurements of inflammation and plaque physiology were performed on sections of the LCI artery at the premenopausal time point. Intimal area containing collagen was significantly increased in *H*. *pylori-*infected animals (Fig 11A and Table 1), while mineralization was modestly increased (Fig 11B and Table 1). Macrophage populations were increased by more than two fold in *H*. *pylori-*infected monkeys as measured by both immunohistochemistry (HAM56; Table 1) and CD68 expression (Fig 11C and Table 1). ICAM1, IL-6 and expression of the T cell markers CD3 and CD4 were significantly increased and expression of the cytokine MCP-1 tended to be higher in infected animals (Fig 11 and Table 1). Our findings indicate an increased inflammatory response due to *H*. *pylori* infection. Overall, the effects of *H*. *pylori* infection were the opposite of the anti-inflammatory effects seen with exposure to the SOY treatment, which decreased macrophage and T cell infiltration, ICAM-1, MCP-1, and IL-6 in these animals[44].

### *H*. *pylori* alters plasma lipid profiles

*H*. *pylori* has been linked to dyslipidemia in humans, as indicated by epidemiological studies and following measurement of plasma lipids before and after *H*. *pylori* eradication [57, 58]. Similarly, we found that *H*. *pylori* infection in monkeys correlated with decreased HDLc, increased non-HDLc (low density and very low density lipoprotein-associated cholesterol), and increased TPC/HDLc (Castelli’s ratio) (all p<0.05; Table 2). In contrast with plaque measurements, the density of *H*. *pylori* in the gastric mucosa (16S and CagA) correlated better with plasma lipids than did *H*. *pylori* density in the artery. Also, the presence of *cagA* did not appear to be an important contributor to dyslipidemia, as indicated by the stronger correlations between plasma lipids and stomach *H*. *pylori* 16S than plasma lipids and artery or stomach *cagA*. This suggests that either local effects of *H*. *pylori* in the stomach or systemic effects of infection are responsible for plasma lipid alterations. Body weight, total cholesterol, and triglycerides were not associated with *H*. *pylori* infection.

### Known viral infections do not influence atherosclerosis in these animals

Human epidemiological studies have noted a relationship between atherosclerosis and viral infections [59]. To explore this possibility, monkeys were tested for Herpes B virus, simian immunodeficiency virus (SIV), measles, Rhesus cytomegalovirus (RhCMV), simian T cell leukemia virus (STLV), types 1 and 2, and simian type D retrovirus (SRV/D). None of the viral infections correlated with the atherosclerosis biomarkers tested in this study.

### Subordinate social status is associated with increased *H*. *pylori* burden

Premenopausal female cynomolgus macaques of low social status are typically at greater risk of atherosclerosis than their dominant counterparts [41]. Could *H*. *pylori* infection influence this risk? We found that subordinate status was significantly associated with increased risk for *H*. *pylori* infection and increased infection density (p< 0.05 using either Fisher’s exact test or Pearson’s Chi-squared test with Yates’ continuity correction). Only 48% of the dominant monkeys were infected with *H*. *pylori* as compared with 78% of the subordinate animals; however, rank did not influence either premenopausal or postmenopausal plaque measurements when *H*. *pylori* infection status was included in the predictive model. Therefore, *H*. *pylori* infection may be a more important contributor to atherosclerosis than social status or, alternatively, status may exert its effects in part through modified resistance to this bacterial infection.

## Discussion

This work is the first study to examine the effects of *H*. *pylori* on development of atherosclerosis in non-human primates and the first to demonstrate that *H*. *pylori* within atherosclerotic plaques are alive. Natural infection with *H*. *pylori* has been documented in both rhesus (*M*. *mulatta*) and cynomolgus macaques [60–62], and rhesus macaques have long been used to study *H*. *pylori* gastric pathogenesis, evolution, and carcinogenesis [60, 63, 64]. However, the potential for natural infection by *H*. *pylori* to influence extragastric pathology in non-human primates had not been explored previously.

Detection of *H*. *pylori* DNA by PCR in human atherosclerotic plaques was first reported by Farsak *et al*. in 2000 and has since been confirmed by a number of other investigators [26–29]; nonetheless, PCR of DNA does not prove the presence of live *H*. *pylori* within plaques. *In situ* hybridization has been routinely used to detect mRNA in paraffin-embedded tissue and is a sensitive and specific method for detecting *H*. *pylori* in gastric biopsies [65, 66]. Our detection of 16S and *cagA* mRNA, combined with corresponding structures matching the size and morphology of *H*. *pylori*, confirm that live *H*. *pylori* inhabit atherosclerotic plaques. The presence of bacterial clusters further suggests that limited bacterial growth occurs within these same plaques. Seeding of heart valves by hematogenously spread oral flora is a well-documented risk and *H*. *pylori* within gastric and oral blood vessels has been previously documented [56, 67], so perhaps it is not surprising that *H*. *pylori* entering the bloodstream can reach the heart and adhere. *H*. *pylori* requires host cholesterol for optimal membrane integrity [68, 69] and the cholesterol-rich atherosclerotic plaques may encourage survival and growth of *H*. *pylori*. The distinction between living or dead *H*. *pylori* within plaques is critical because live *H*. *pylori* can inject bacterial components including the CagA toxin, peptidoglycan, DNA, and metabolites of lipopolysaccharide into the host cell cytoplasm via the type IV secretion apparatus [70, 71]; dead *H*. *pylori* cannot influence host cell signaling in this manner. Therefore, in terms of atherosclerosis *H*. *pylori* is likely to directly activate inflammatory pathways within the atheroma in addition to acting indirectly via distal activity at the site of gastric colonization.

*H*. *pylori* significantly worsened several plaque parameters, including common iliac artery intimal thickness and area in the same animals evaluated pre- and postmenopausally. Intimal collagen density and mineralization were also affected. Although the majority of macaques were infected with a mixture of *cagA-*positive and *cagA-*negative *H*. *pylori* strains, we were able to examine the contributions of *cagA* by determining whether LCX artery measurements were more greatly influenced by total bacterial density (as determined by 16S) or by *cagA-*positive bacterial density. That analysis revealed that *cagA-*positive strains were more atherogenic than *cagA-*negative strains. Moreover, *H*. *pylori* densities found within the arteries had a greater impact than bacterial density in the stomach. Though not conclusive, these data suggest that plaque development is due to local effects of *cagA-*positive *H*. *pylori*. Notably, these findings are consistent with epidemiological studies conducted in humans. While early epidemiological studies investigating the role of *H*. *pylori* in cardiovascular diseases yielded mixed results [17–20], recent studies that differentiate between *cagA-*positive vs. *cagA-*negative strains have yielded more consistent positive associations [24, 72–74]. Interestingly, Franceschi *et al* reported cross-reactivity between CagA-specific antibodies and artery tissue [75]. This immunoreactivity was assumed to be due to molecular mimicry, yet the authors did not determine whether antibodies specific for other *H*. *pylori* proteins reacted with the tissue or prove that the “cross-reactive” antigen was of human origin. In light of the evidence shown here, it is likely that CagA itself was being detected.

Another intriguing finding of this study was that the densities of *H*. *pylori* in the gastric mucosa were highly correlated with the densities in the coronary artery plaques. Gastric colonization density is known to vary among individuals and is believed to be related to both the *H*. *pylori* strain and the type of immune response generated by the host [76–78]. We could not determine whether colonization density varied over time, but the high correlation between postmenopausal bacterial density and both pre- and postmenopausal plaque measurements may suggest that colonization density is stable. Additional studies will be needed to determine how *H*. *pylori* densities in both locations change over time and whether gastric biopsies could be used to estimate the risk of severe atherosclerosis.

In addition to affecting plaque measurements, *H*. *pylori* infection was associated with increases in some arterial inflammatory markers, providing a possible mechanism for *H*. *pylori-*mediated atherosclerosis exacerbation. VCAM-1, ICAM-1, and MCP-1 all contribute to recruitment of macrophages to plaques [79, 80] and all tended to be increased in *H*. *pylori-*infected macaques. IL-6, which induces ICAM-1 [81], was also elevated by *H*. *pylori*. Our findings are consistent with *in vitro* induction of P-selectin, E-selectin, VCAM-1, and ICAM-1 and enhanced transendothelial migration of activated CD4^+^ and CD8^+^ T cells in human umbilical vein endothelial cell cultures following *H*. *pylori* exposure [82, 83]. We similarly found elevated plaque CD4^+^ T cells, which are pro-atherogenic [84], in *H*. *pylori-*positive animals. Others have reported that *H*. *pylori* induces MCP-1, IL-6, and ICAM-1 in various cell types [85–88] and endothelial ICAM1 expression strongly correlates with the presence of *H*. *pylori* DNA in human atherosclerotic plaques [89]. Furthermore, Figura *et al*. report that acute coronary artery disease patients infected with *cagA-*positive *H*. *pylori* have higher circulating IL-6 levels than uninfected patients [90]. Our results confirm and expand upon previous results, since we have established that live *H*. *pylori* within atherosclerotic lesions can directly interact with macrophages and/or endothelial cells to induce cytokine and adhesion molecule production.

Clinical studies have also reported decreased HDL and increased LDL in association with *H*. *pylori*, and some studies have found reversal of dyslipidemia following *H*. *pylori* eradication [57, 58, 91], although not all studies have found such a link [92]. One Japanese study found *H*. *pylori-*associated dyslipidemia in male, but not female subjects [93]; we clearly saw dyslipidemia in female, postmenopausal macaques. The varied findings in human studies likely result from the considerable differences among humans in terms of diet, lifestyle, genetics, and many other factors, many of which were controlled for in our monkey study. Some studies have linked dyslipidemia with atrophic gastritis in *H*. *pylori-*positive patients [94, 95]. This is consistent with our observation that plasma lipid changes were more closely related to gastric *H*. *pylori* densities than arterial densities.

Atherosclerosis development is delayed in women compared with men and estrogens likely mediate this effect based on studies showing that premenopausal oophorectomy or premature ovarian failure are associated with increased coronary heart disease risk, an effect that can be mitigated by treatment with exogenous estrogen in younger, but not older women [96]. Soy phytoestrogens improve cardiovascular disease biomarkers in some human studies involving men and post-menopausal women [97]. This may be in part due to the effects of soy on ER-α and ER-β, which are expressed in arteries [48]. *H*. *pylori* increased atheroma measurements both pre- and post-menopausally and generally counteracted the beneficial effects of SOY on inflammatory markers in cynomolgus macaques (reported by Walker *et al* [48]); however, unlike the SOY diet, *H*. *pylori* did not significantly alter expression of ER-α or ER-β. Therefore, the increased risk associated with *H*. *pylori* infection seems unaffected by estrogen.

The differing *H*. *pylori* burdens among dominant and subordinate animals have not been previously reported. Female subordinate macaques are under considerably more stress than dominant females and typically develop more atherosclerosis than their dominant counterparts when consuming an atherogenic diet [41], an effect that co-occurs with substantial ovarian disruption [96]. In humans, lower socioeconomic status positively correlates with *H*. *pylori* prevalence, presumably due to poor hygiene and overcrowding [98]. Although human socioeconomic status is not homologous to monkey dominance relationships [40], subordinate female macaques are directly stressed as a result of being physically harassed by their dominant counterparts during competitive interactions over food, space, and preferred grooming partners [99]. Moreover, in the small group context used in the study reported here, dominant animals demonstrably outcompete subordinates for fresh food and water and desirable perching locations, potentially exposing subordinates to more fecal contamination as they consume food and water that drop or spill to the ground. Such contamination, in turn, is one proposed transmission route for *H*. *pylori* [100]. Of note, several large epidemiological studies report an increased risk of cardiovascular disease in humans with low socioeconomic status. Although, again, human socioeconomic status differs conceptually from monkey dominance status [40], the increased *H*. *pylori* burden in humans with low socioeconomic status could contribute to higher atherosclerosis risk in this group.

The primary weakness of this study is that we are not certain when the animals became infected or whether bacterial prevalence changed over time. Humans most often become colonized during childhood, but transmission can occur later in life, particularly between spouses [101–103]. In rhesus macaques, *H*. *pylori* transmission occurs within 90 days via social contact, rather than fomites, but some animals remain uninfected, possibly due to host genetic factors [104, 105]. *H*. *pylori* infection persists for at least a decade in Rhesus macaques [63] indicating that infection is as persistent in non-human primates as it is in humans. If the animals in this study were not previously infected, then transmission likely occurred soon after assignment to social groups. The substantial correlations between postmenopausal *H*. *pylori* status and premenopausal plaque parameters further indicate that animals were infected before the study midpoint.

For the first time, this study conclusively demonstrated that live *H*. *pylori* can reside within atherosclerotic plaques and that *H*. *pylori* is likely to have local effects on plaque size. Furthermore, *H*. *pylori* influenced macrophage infiltration, collagen deposition, ICAM1 expression, and plasma lipids. The effects of *H*. *pylori* on plaque parameters appeared to be primarily due to local effects of *cagA-*positive *H*. *pylori*, whereas changes in plasma lipids related to gastric infection by either *cagA-*positive or–negative strains. *H*. *pylori* status was relevant during both the premenopausal and postmenopausal phases as demonstrated by correlations between *H*. *pylori* infection and iliac artery plaque parameters measured at both ovariectomy and necropsy. Given the many similarities in atherogenesis between humans and non-human primates, future inclusion of *cagA-*positive *H*. *pylori* status in human epidemiological and clinical atherosclerosis studies is warranted.

## Supporting information

S1 Table Raw data used in this study. (XLSX)
